# Invasion Patterns and Long-Term Clinical Outcomes of Diffuse Tenosynovial Giant Cell Tumor of the Ankle Joint

**DOI:** 10.7759/cureus.56148

**Published:** 2024-03-14

**Authors:** Kumiko Yotsuya, Yoji Shido, Yukihiro Matsuyama

**Affiliations:** 1 Department of Orthopedic Surgery, Hamamatsu University School of Medicine, Hamamatsu, JPN

**Keywords:** invasion patterns, recurrence rate, ankle joint, pigmented villonodular synovitis, diffuse type tenosynovial giant cell tumor

## Abstract

Background: The invasion patterns and long-term outcomes of diffuse tenosynovial giant cell tumor (D-TSGCT) of the ankle joint remain unclear.

Methods: Seven patients who visited our department between 2011 and 2023 and were diagnosed with D-TSGCT of the ankle joint by contrast-enhanced MRI and a pathological diagnosis were included. The invasion patterns of ankle D-TSGCT on MRI were investigated. The recurrence rate and clinical symptoms were examined in five patients followed up for more than seven years after total resection.

Results: In seven patients (1 male/6 females, mean age 37.0±16.6 years, range 15-57 years) with D-TSGCT of the ankle joint, contrast-enhanced MRI at the initial presentation showed invasion within the ankle joint, extending along the tendon sheath, within the talocalcaneal joint, and in the tarsal sinus in 100% of cases, around the deltoid ligament in 86%, within the plantar surface in 43%, invasion of the interosseous membrane in 57%, around the Achilles tendon in 29%, and scalloping on the talocrural joint in 43%. The mean time from mass awareness to the first visit was 51.9±80.0 months (range 1-240 months). Gross total resection, defined as the removal of all tumors as gauged by MRI, was initially performed on 6/7 patients. One patient underwent partial resection of only the anterior part of the tumor. Of the six cases in which gross total resection was performed, 5 had long-term follow-up of more than seven years post-operatively, and one case is still only one year post-operatively. The long-term results of five patients followed for more than seven years after total resection were as follows: a mean follow-up period of 125 months (range 89-171 months), a 100% recurrence rate, a mean time to recurrence of 27.5±19.2 months (range 7-60 months), and a 16% reoperation rate. In the last follow-up, osteoarthritic changes were observed radiographically in 2/5 patients (40%), both of whom had scalloping of the talocrural joint on MRI at the time of the initial diagnosis. Four of the five patients (80%) had no clinical symptoms in the last follow-up.

Conclusion: Ankle D-TSGCT presents with a strong local infiltrative pattern inside and outside the ankle joint along the tendon sheath, radical resection may be difficult, and the recurrence rate may be higher than previously reported. On the other hand, there are many cases that remain free of clinical symptoms in the long term after recurrence, and surgical indications for ankle D-TSGCT need to consider function preservation as well as recurrence rates.

## Introduction

Diffuse tenosynovial giant cell tumor (D-TSGCT), formerly known as pigmented villonodular synovitis, is a benign synovial tumor involving joints, tendon sheaths, and joint capsules, with an estimated incidence of 4-9.2 per million persons per year [1-3]. Its estimated incidence is 64% in the knee joint, 14% in the ankle joint, 10% in the hip joint, 5% in the foot, and 1% in the shoulder joint [4]. Although surgical resection is the primary treatment, it is difficult to remove the lesion completely, the postoperative recurrence rate is high, and osteochondral destruction leads to long-term functional impairment [5,6].

The ankle joint involves more anatomically complex structures than the knee and hip joints, with many ligaments, tendon sheaths, and adjacent joints around a narrow joint cavity. Although the recurrence rate of D-TSGCT arising in the foot and ankle joints was previously shown to be 21-61%, which is lower than that in the knee and hip joints, the majority of studies reported the foot and ankle joints together, and the average follow-up period was only between 37.9 months and 7.7 years [7-9]. Therefore, the invasion patterns and long-term clinical outcomes of D-TSGCT of the ankle joint have not been adequately investigated. Since the ankle joint is a weight-bearing joint, it is necessary to examine the long-term clinical outcomes of the ankle joint separately from those of the toes.

Therefore, we herein investigated invasion patterns on MRI in seven cases of ankle D-TSGCT. We also examined the long-term clinical outcomes of five cases and six surgeries that were followed up for more than seven years (mean follow-up of 10 years and five months) after surgery.

## Materials and methods

**Figure 1:**
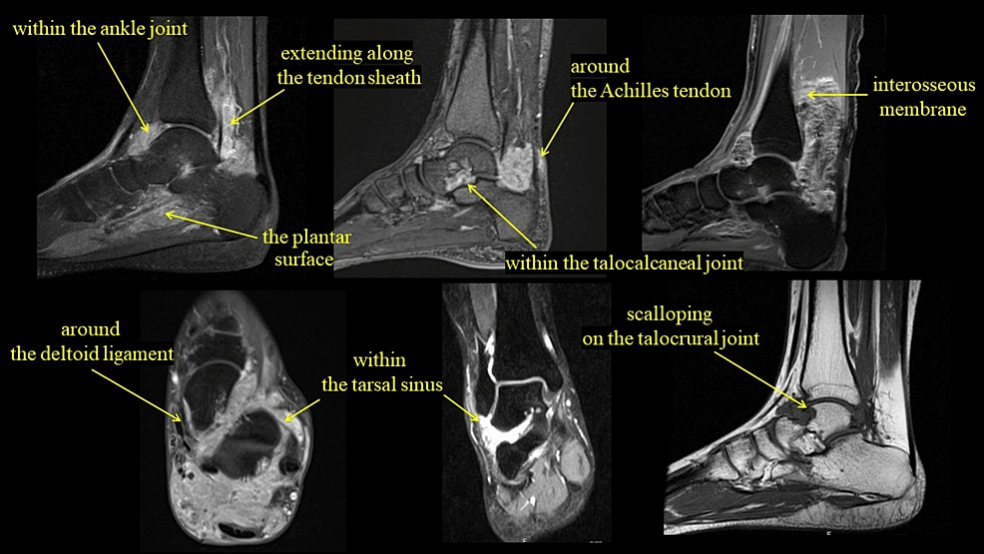
Location of the tumor invasion site

We conducted a survey based on previous electronic medical record data at a single institution (Hamamatsu University School of Medicine). All patients who visited our department between January 2011 and December 2023 and were diagnosed with D-TSGCT of the ankle joint by contrast-enhanced MRI and a pathological diagnosis were included. Seven patients were included in the study (1 male/6 females, mean age 37.0±16.6 years). We investigated the site of tumor invasion on contrast-enhanced MRI at the time of the initial diagnosis, and the time from mass awareness to the initial diagnosis. Tumor invasion sites were divided into the following areas: within the ankle joint, extending along the tendon sheath, within the talocalcaneal joint, within the tarsal sinus, around the deltoid ligament, plantar surface, and the interosseous membrane, around the Achilles tendon, and scalloping on the talocrural joint (Figure 1).

Gross total resection is defined as the removal of all tumors, as gauged by MRI. Of these seven cases, we performed initial gross total tumor resection in six cases. We have indicated gross total resection in all cases, but in the seventh case, a national-level volleyball player, we prioritized preservation of his ankle joint function and early return to sport and decided on a partial resection. Of the six cases in which gross total resection was performed, reoperation (gross total resection) was performed in one case that recurred locally and was symptomatic. Of the six cases in which gross total resection was performed, 5 had a long-term follow-up of more than seven years postoperatively, and one case (sixth case) is still only one year postoperatively. There were six operations in five patients who could be followed up for more than seven years after surgery. These five patients and six gross total tumor resection surgeries with a follow-up of more than seven years after surgery were investigated for the following: recurrence, time to recurrence, osteoarthritic changes on plain radiographs in the last follow-up, and clinical symptoms in the last follow-up.

Since the present study was conducted on a case series using only historical electronic medical record data, Ethics Committee approval was not required at this hospital. Written informed consent was obtained from all patients for publication and any accompanying images.

## Results

In seven patients (1 male/6 females, mean age 37.0±16.6 years, range 15-57 years) with D-TSGCT of the ankle joint, contrast-enhanced MRI at the initial presentation showed invasion within the ankle joint, extending along the tendon sheath, within the talocalcaneal joint, and in the tarsal sinus in 100% of cases, around the deltoid ligament in 86%, within the plantar surface in 43%, invasion of the interosseous membrane in 57%, around the Achilles tendon in 29%, and scalloping on the talocrural joint in 43% (Table 1, Table 2). The mean time from awareness of symptoms such as swelling, limited range of movement, or pain to the first visit was 51.9±80.0 months (range 1-240 months). Gross total resection was initially performed on 6/7 patients, and one patient underwent partial resection of only the anterior part of the tumor (Table 1).

**Table 1:** Patient backgrounds and results for all cases

Case No.	Sex	Age (years)	Time since mass awareness (months)	Scalloping on the talocrural joint	Operation
1	F	46	4	+	Gross total resection
2	F	23	60	−	Gross total resection
3	F	17	48	+	Gross total resection
4	F	57	3	−	Gross total resection
5	F	52	240	−	Gross total resection
6	F	49	7	−	Gross total resection
7	M	15	1	+	Partial resection

**Table 2:** Results of contrast-enhanced MRI of tumor invasion sites in seven cases

Invasion sites	n	Percentage
Within the ankle joint	7/7	100%
Extending along the tendon sheath	7/7	100%
Within the talocalcaneal joint	7/7	100%
Within the tarsal sinus	7/7	100%
Around the deltoid ligament	6/7	86%
Invasion of the interosseous membrane	4/7	57%
Scalloping on the talocrural joint	3/7	43%
The plantar surface	3/7	43%
Around the Achilles tendon	2/7	29%

Of the six cases in which gross total resection was performed, 5 had long-term follow-up of more than seven years postoperatively, and one case is still only one year postoperatively. The long-term results of six surgeries performed on five patients followed for more than seven years after total resection were as follows: a mean follow-up period of 125 months (range 89-171 months), a 100% recurrence rate in the same location as the primary lesion, a mean time to recurrence of 27.5±19.2 months (range 7-60 months), and a 16% reoperation rate. In the last follow-up, osteoarthritic changes were observed radiographically in 2/5 patients (40%), both of whom had scalloping of the talocrural joint on MRI at the time of the initial diagnosis. Four of the five patients (80%) had no clinical symptoms in the last follow-up (Table 3).

**Table 3:** Results of six surgeries on five patients who underwent gross total tumor resection ROM: Range of motion; KL: Kellgren-Lawrence grade

Case No.	Age at surgery (year)	Preoperative symptoms	Postoperative follow-up period (months)	Recurrence	Period until recurrence (months)	Reoperation	Clinical symptoms in the final observation	Osteoarthritis on radiographs in the final observation
1	46	Pain, limited ROM, Swelling	148	+	36	+	Pain during loading	KL-4
1'	48	Pain, limited ROM	118	+	7	−		
2	23	Pain, Swelling	120	+	21	−	−	−
3	17	Swelling	171	+	29	−	−	KL-3
4	57	Swelling	104	+	12	−	−	−
5	52	Swelling	89	+	60	−	−	−

## Discussion

The present results showed that D-TSGCT of the ankle joint showed a strong local infiltrative pattern along the tendon sheath both inside and outside the ankle joint on MRI. The rate of involvement of the internal and external ankle joints along the tendon sheath, the talocalcaneal joint, and the tarsal sinus was as high as 100%. Until recently in our department, all cases were treated by gross total resection; however, all cases recurred, and radical total resection was considered to be difficult. Although surgery was aggressively performed to remove the recurrent lesion in case 1, it recurred and the patient eventually developed osteoarthritis of the ankle. On the other hand, many cases remained free of clinical symptoms for a long period of time without surgery after recurrence (Table 3).

The recurrence rate of the foot and ankle joints was previously reported to be 21-61%, which is lower than that of the knee and hip joints; however, in the present study, the recurrence rate was 100% after a mean follow-up of 125.0±29.9 months for six ankle D-TSGCT surgeries [7-9]. The reasons for the higher recurrence rate in the present study are that the majority of previous studies combined ankle and foot surgical outcomes, including some cases of toe amputation, in addition to the longer postoperative follow-up period in this study. The present results revealed that ankle D-TSGCT invaded the ankle joint both internally and externally along the tendon sheath and was often present in the tarsal sinus and talocalcaneal joint; therefore, radical resection may be difficult and the actual recurrence rate in surgical treatment may be higher than previously reported. The ankle joint is anatomically characterized by a narrower joint cavity than the hip or knee joint, with many tendon sheaths in contact with the ankle joint capsule. Therefore, D-TSGCT of the ankle joint may be less likely to remain within the joint cavity and more likely to spread across the compartment into the lower leg and foot.

Siegel performed a meta-analysis of 212 cases (25 references) of D-TGCT of the ankle and foot and reported a mean follow-up of 37.9±27.4 months, a recurrence rate of 21%, with the time to recurrence ranging between 3 and 144 months, and 86% within 5 years of treatment [9]. In the present study, the mean time for postoperative recurrence was 27.5±19.2 months (range 7-60 months) and there was one case of recurrence at five years postoperatively, suggesting that D-TSGCT requires a long-term postoperative follow-up of at least five years.

Barnett conducted a retrospective analysis of 123 TSGCT cases of the ankle and foot and found that patients with pain at presentation and those with erosive changes on MRI at presentation were more likely to have persistent postoperative pain (p <0.001), with 57.1% of patients with both pain and erosive changes preoperatively having postoperative pain [7]. In the present study, two patients with osteoarthritic changes on MRI in the last follow-up also had preoperative scalloping of the talocrural joint, while only one had clinical symptoms. In addition, one of the two patients with preoperative pain had persistent pain in the last follow-up. Preoperative joint scalloping and preoperative pain may predict the long-term clinical course. In D-TSGCT of the knee, ankle, and hip joints, performing a bone graft or cement filling of the defect at the same time as tumor resection is considered when subchondral bone lesions, such as bone cysts or bone defects, are present preoperatively [10]. The presence of preoperative articular bone lesions may indicate long-term osteoarthritis, which may require informing the patient and considering additional procedures depending on the size of the subchondral bone lesion.

Since TSGCT is a benign tumor, treatment may need to focus not only on the local recurrence rate but also on preserving the function of the affected joint. Ota reported that arthroscopic surgery for D-TSGCT of the knee joint was useful for preserving joint function; however, arthroscopic surgery may have a higher recurrence rate than open surgery [11]. Furthermore, repeat surgery for local recurrence significantly increased the progression of osteochondral destruction [12]. Open surgery is associated with a number of complications, such as joint contracture and sympathetic dystrophy, while repeated surgery for local recurrence has been reported to increase postoperative complications [13,14]. In the present study, one patient who underwent additional surgery for local recurrence eventually developed osteoarthritis and clinical symptoms (case 1), whereas the others remained free of clinical symptoms in the long term without surgery after recurrence. Therefore, the indication for surgery for D-TSGCT of the ankle joint needs to consider not only the oncological outcome but also functional preservation. Arthroscopic surgery for D-TSGCT of the ankle and arthroscopic surgery of the toe were shown to have a higher recurrence rate than open surgery [8]. However, long-term osteochondral changes and clinical symptoms have not been reported and, thus, future studies are needed on long-term outcomes in consideration of functional aspects. Until recently in our department, all cases underwent gross total resection; however, in case 7, partial resection was performed with priority given to preserving ankle joint function even with the increased risk of local recurrence. Although preoperative symptoms improved in the short term, long-term tumor outcomes, osteochondral destruction, and clinical symptoms need to be followed up in the future.

Baniel reported that D-TSGCT was difficult to completely remove and that postoperative radiotherapy of 1.8-2.5 Gy/dose × 4 to a total of 34-36 Gy achieved local control and the preservation of joint function and prevented the risk of recurrence and recurrent lesions [15]. Postoperative radiotherapy was not performed in our patients because the patients did not want it, but it may be a treatment option for postoperative recurrence. The effects of several systemic therapies, including CSF-1 receptor monoclonal antibodies and tyrosine kinase inhibitors, have recently been investigated and are expected to be expanded for future indications [16,17].

A limitation of this study is the small number of ankle D-TSGCT cases examined in a single-center study.

## Conclusions

We found in this investigation that ankle D-TSGCT presents with a strong local infiltrative pattern inside and outside the ankle joint along the tendon sheath. Radical resection may be difficult, and the recurrence rate may be extremely high. On the other hand, there were many cases that remained free of clinical symptoms in the long term after recurrence. Surgical indications for ankle D-TSGCT need to consider function preservation as well as recurrence rates.

## References

[1] Ehrenstein V, Andersen SL, Qazi I, Sankar N, Pedersen AB, Sikorski R, Acquavella JF (2017). Tenosynovial giant cell tumor: incidence, prevalence, patient characteristics, and recurrence. A registry-based cohort study in Denmark. J Rheumatol.

[2] Mastboom MJ, Verspoor FG, Verschoor AJ (2017). Higher incidence rates than previously known in tenosynovial giant cell tumors. Acta Orthop.

[3] Myers BW, Masi AT (1980). Pigmented villonodular synovitis and tenosynovitis: a clinical epidemiologic study of 166 cases and literature review. Medicine (Baltimore).

[4] Mastboom MJ, Palmerini E, Verspoor FG (2019). Surgical outcomes of patients with diffuse type tenosynovial giant-cell tumors: an international, retrospective, cohort study. Lancet Oncol.

[5] Ma X, Shi G, Xia C, Liu H, He J, Jin W (2013). Pigmented villonodular synovitis: a retrospective study of seventy five cases (eighty one joints). Int Orthop.

[6] Ottaviani S, Ayral X, Dougados M, Gossec L (2011). Pigmented villonodular synovitis: a retrospective single-center study of 122 cases and review of the literature. Semin Arthritis Rheum.

[7] Barnett JR, Rudran B, Khan A (2023). Outcomes of tenosynovial giant cell tumor of the foot and ankle. Foot Ankle Int.

[8] Spierenburg G, Lancaster ST, van der Heijden L (2021). Management of tenosynovial giant cell tumour of the foot and ankle. Bone Joint J.

[9] Siegel M, Bode L, Südkamp N, Kühle J, Zwingmann J, Schmal H, Herget GW (2021). Treatment, recurrence rates and follow-up of tenosynovial giant cell tumor (TGCT) of the foot and ankle-a systematic review and meta-analysis. PLoS One.

[10] Jamshidi K, Sharifi Dalooei SM, Bagherifard A, Mirzaei A (2023). Total synovectomy and bone grafting/cementation after curettage of the bone lesion in diffuse‏ type ‏of tenosynovial‏ giant cell tumor‎: a retrospective cohort study. Arch Bone Jt Surg.

[11] Ota T, Nishida Y, Ikuta K (2021). Tumor location and type affect local recurrence and joint damage in tenosynovial giant cell tumor: a multi-center study. Sci Rep.

[12] Nishida Y, Tsukushi S, Nakashima H (2012). Osteochondral destruction in pigmented villonodular synovitis during the clinical course. J Rheumatol.

[13] Chin KR, Barr SJ, Winalski C, Zurakowski D, Brick GW (2002). Treatment of advanced primary and recurrent diffuse pigmented villonodular synovitis of the knee. J Bone Joint Surg Am.

[14] Sharma V, Cheng EY (2009). Outcomes after excision of pigmented villonodular synovitis of the knee. Clin Orthop Relat Res.

[15] Baniel C, Yoo CH, Jiang A (2023). Long-term outcomes of diffuse or recurrent tenosynovial giant cell tumor treated with postoperative external beam radiation therapy. Pract Radiat Oncol.

[16] Tap WD, Gelderblom H, Palmerini E (2019). Pexidartinib versus placebo for advanced tenosynovial giant cell tumour (ENLIVEN): a randomised phase 3 trial. Lancet.

[17] Robert M, Farese H, Miossec P (2022). Update on tenosynovial giant cell tumor, an inflammatory arthritis with neoplastic features. Front Immunol.

